# Morpholin-4-ium hydrogen l-tartrate monohydrate

**DOI:** 10.1107/S1600536811055620

**Published:** 2012-01-07

**Authors:** T. Kishore Kumar, D. Prem Anand, S. Selvakumar, S. Pandi, M. NizamMohideen

**Affiliations:** a Department of Physics, Presidency College (Autonomous), Chennai 600 005, India; b Department of Physics, St. Xavier’s College, Palayamkottai 627 002, India; c Department of Physics, Govt. Arts College (Autonomous), Chennai 600 035, India; d Department of Physics, The New College (Autonomous), Chennai 600 014, India

## Abstract

In the title compound, C_4_H_10_NO^+^·C_4_H_5_O_6_^−^·H_2_O, the morpholine ring adopts a chair conformation. In the crystal, the tartrate anions are linked *via* O—H⋯O hydrogen bonds, forming chains propagating along [101]. These chains are linked *via* N—H⋯O and O—H⋯O hydrogen bonds, involving the morpholinium cation and the water molecule, forming a three-dimensional network.

## Related literature

For the biological activity of morpholine derivatives, see: Lan *et al.* (2010 ▶); Raparti *et al.* (2009 ▶). For standard bond lengths, see: Allen *et al.* (1987 ▶). For related studies on co-crystals of amino derivatives, see: Fu *et al.* (2010 ▶); Aminabhavi *et al.* (1986 ▶). For puckering parameters, see: Cremer & Pople (1975 ▶) and for asymmetry parameters, see: Nardelli (1983 ▶).
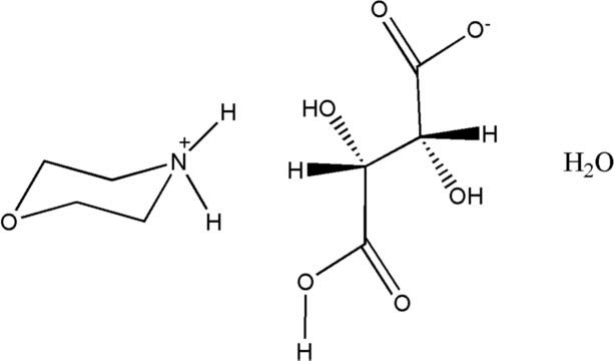


## Experimental

### 

#### Crystal data

C_4_H_10_NO^+^·C_4_H_5_O_6_^−^·H_2_O
*M* *_r_* = 255.23
Triclinic, 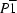

*a* = 7.6260 (3) Å
*b* = 8.2408 (3) Å
*c* = 10.1674 (4) Å
α = 98.462 (1)°
β = 106.282 (1)°
γ = 104.807 (1)°
*V* = 576.25 (4) Å^3^
*Z* = 2
Mo *K*α radiation
μ = 0.13 mm^−1^
*T* = 293 K
0.25 × 0.20 × 0.20 mm

#### Data collection

Bruker Kappa APEXII CCD diffractometer
Absorption correction: multi-scan (*SADABS*; Bruker, 2004 ▶) *T*_min_ = 0.968, *T*_max_ = 0.974
15849 measured reflections
3977 independent reflections
3218 reflections with *I* > 2σ(*I*)
*R*_int_ = 0.022

#### Refinement

*R*[*F* ^2^ > 2σ(*F* ^2^)] = 0.038
*wR*(*F* ^2^) = 0.113
*S* = 1.05
3977 reflections
182 parameters
H atoms treated by a mixture of independent and constrained refinement
Δρ_max_ = 0.42 e Å^−3^
Δρ_min_ = −0.21 e Å^−3^

### 

Data collection: *APEX2* (Bruker, 2004 ▶); cell refinement: *SAINT* (Bruker, 2004 ▶); data reduction: *SAINT*; program(s) used to solve structure: *SHELXS97* (Sheldrick, 2008 ▶); program(s) used to refine structure: *SHELXL97* (Sheldrick, 2008 ▶); molecular graphics: *ORTEP-3* (Farrugia, 1997 ▶) and *PLATON* (Spek, 2009 ▶); software used to prepare material for publication: *SHELXL97*.

## Supplementary Material

Crystal structure: contains datablock(s) global, I. DOI: 10.1107/S1600536811055620/lx2207sup1.cif

Structure factors: contains datablock(s) I. DOI: 10.1107/S1600536811055620/lx2207Isup2.hkl

Additional supplementary materials:  crystallographic information; 3D view; checkCIF report

## Figures and Tables

**Table 1:** Hydrogen-bond geometry (Å, °)

*D*—H⋯*A*	*D*—H	H⋯*A*	*D*⋯*A*	*D*—H⋯*A*
N1—H1*B*⋯O3^i^	0.856 (17)	2.036 (17)	2.8430 (11)	156.6 (15)
N1—H1*A*⋯O1*W*	0.888 (18)	1.888 (18)	2.7583 (13)	166.1 (16)
O1*W*—H1*W*⋯O4^ii^	0.825 (19)	2.013 (19)	2.8173 (11)	164.6 (18)
O1*W*—H2*W*⋯O5^iii^	0.848 (19)	1.921 (19)	2.7542 (11)	167.2 (17)
O2—H2*A*⋯O6^iv^	0.958 (19)	1.584 (19)	2.5412 (10)	177.3 (17)
O3—H3*A*⋯O5^v^	0.911 (17)	1.742 (17)	2.6398 (9)	167.9 (15)
O4—H4*A*⋯O7	0.868 (16)	1.939 (16)	2.7818 (10)	163.4 (14)

Symmetry codes: (i) 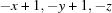; (ii) 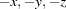; (iii) 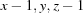; (iv) 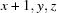; (v) 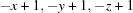.

**Fig. 1.**
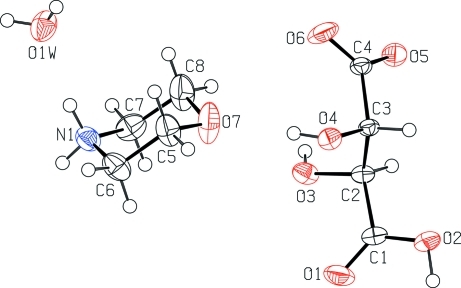
The molecular structure of the title compound with the atom numbering scheme. Displacement ellipsoids are drawn at the 50% probability level. H atoms are presented as small spheres of arbitrary radius.

**Fig. 2.**
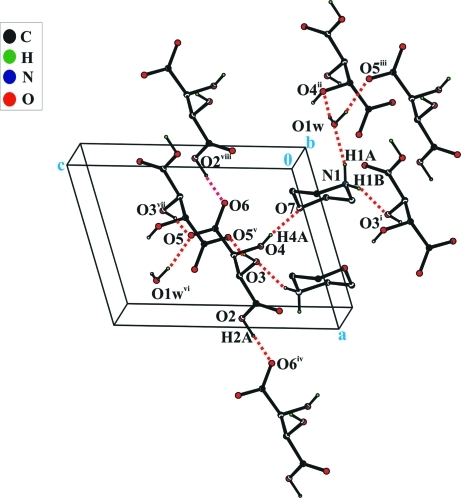
A view of the N–H···O and O–H···O hydrogen bonds (dotted lines) in the crystal structure of the title compound. H atoms non-participating in hydrogen-bonding were omitted for clarity. [Symmetry codes: (i) - x+1, - y + 1, - z ; (ii) - x, - y, - z ; (iii) x - 1, y, z - 1; (iv) x + 1, y, z ; (v) - x + 1, - y + 1, - z + 1; (vi) x + 1, y, z + 1; (vii) - x + 1, - y + 1, - z + 1; (viii) x - 1, y, z.]

## supplementary crystallographic information

### Comment

Morpholine derivatives possess anticancer and antimicrobial (Lan *et al.*, 2010; Raparti *et al.*, 2009) activities. The amino derivatives have found wide range of applications in material science, such as magnetic, fluorescent and dielectric behaviors, and there has been an increasing interest in the preparation of amino co-crystal compounds (Aminabhavi *et al.*, 1986; Fu, *et al.* 2010). Here we report the crystal structure of the title compound (Fig. 1).

All geometric parameters are in the normal ranges (Allen *et al.*, 1987). The morpholine ring (N1/O7/C5–C8) adopts an almost perfect normal chair conformation having a total puckering amplitude, Q_T_ of 0.568 (2) Å and [θ = 176.2 (2) and φ = 180.1 (2)°] (Cremer & Pople, 1975), and the lowest displacement asymmetry parameters Δ_S_(O7/N1) is 0.11 (2)° (Nardelli, 1983). The crystal structure of the title compound is characterized by intermolecular bifurcated N–H···O and O–H···O hydorgen bond (Table. 1 and Fig. 2). The morpholinium cations and tartrate anions are linked through intermolecular bifurcated N–H···O and O–H···O hydrogen bonds, forming a chain. The chains and water molecules interact, generating an O–H···O hydrogen-bonded layer.

### Experimental

Cold absolute methanol (60 ml) was added to L-tartaric acid (2.94 g, 19.62mmol). The acid was dissolved by heating the mixture on a hot plate with stirring maintained at a temperature of 358 K. The solution was cooled to 298 K and morpholine (1.70 g, 19.62 mmol) was added dropwise. The product was precipitated out of the solution as a white tiny seed crystals by spontaneous nucleation (78.3 %, m.p. 441-442 K). Single crystals suitable for X-ray diffraction were recrystallized ethl alcohol.

### Refinement

The H atoms bonded to O1w were located a different Fourier map and refined freely. All other H atoms were positioned geometrically, with C–H = 0.93 and N–H = 0.89Å constrained to ride on their parent atoms, with U_iso_(H) = xU_eq_(C, N), where x = 1.5 for methyl H and x = 1.2 for all other H atoms.

### Crystal data

**Table:**

C_4_H_10_NO^+^·C_4_H_5_O_6_^−^·H_2_O	*Z* = 2
*M**_r_* = 255.23	*F*(000) = 272
Triclinic, *P*1	*D*_x_ = 1.471 Mg m^−^^3^
Hall symbol: -P 1	Mo *K*α radiation, λ = 0.71073 Å
*a* = 7.6260 (3) Å	Cell parameters from 6973 reflections
*b* = 8.2408 (3) Å	θ = 2.6–31.9°
*c* = 10.1674 (4) Å	µ = 0.13 mm^−^^1^
α = 98.462 (1)°	*T* = 293 K
β = 106.282 (1)°	Block, colourless
γ = 104.807 (1)°	0.25 × 0.20 × 0.20 mm
*V* = 576.25 (4) Å^3^	

### Data collection

**Table:**

Bruker Kappa APEXII CCD diffractometer	3977 independent reflections
Radiation source: fine-focus sealed tube	3218 reflections with *I* > 2σ(*I*)
graphite	*R*_int_ = 0.022
Detector resolution: 10.0 pixels mm^-1^	θ_max_ = 32.0°, θ_min_ = 2.2°
ω and φ scan	*h* = −11→11
Absorption correction: multi-scan (*SADABS*; Bruker, 2004)	*k* = −12→12
*T*_min_ = 0.968, *T*_max_ = 0.974	*l* = −15→15
15849 measured reflections	

### Refinement

**Table:**

Refinement on *F*^2^	Primary atom site location: structure-invariant direct methods
Least-squares matrix: full	Secondary atom site location: difference Fourier map
*R*[*F*^2^ > 2σ(*F*^2^)] = 0.038	Hydrogen site location: difference Fourier map
*wR*(*F*^2^) = 0.113	H atoms treated by a mixture of independent and constrained refinement
*S* = 1.05	*w* = 1/[σ^2^(*F*_o_^2^) + (0.0609*P*)^2^ + 0.0699*P*] where *P* = (*F*_o_^2^ + 2*F*_c_^2^)/3
3977 reflections	(Δ/σ)_max_ < 0.001
182 parameters	Δρ_max_ = 0.42 e Å^−^^3^
0 restraints	Δρ_min_ = −0.21 e Å^−^^3^

### Special details

**Table:**

Geometry. All esds (except the esd in the dihedral angle between two l.s. planes) are estimated using the full covariance matrix. The cell esds are taken into account individually in the estimation of esds in distances, angles and torsion angles; correlations between esds in cell parameters are only used when they are defined by crystal symmetry. An approximate (isotropic) treatment of cell esds is used for estimating esds involving l.s. planes.
Refinement. Refinement of F^2^ against ALL reflections. The weighted R-factor wR and goodness of fit S are based on F^2^, conventional R-factors R are based on F, with F set to zero for negative F^2^. The threshold expression of F^2^ > 2sigma(F^2^) is used only for calculating R-factors(gt) etc. and is not relevant to the choice of reflections for refinement. R-factors based on F^2^ are statistically about twice as large as those based on F, and R- factors based on ALL data will be even larger.

### Fractional atomic coordinates and isotropic or equivalent isotropic displacement parameters (Å^2^)

**Table:**

	*x*	*y*	*z*	*U*_iso_*/*U*_eq_	
O1	0.94402 (12)	0.37187 (11)	0.27498 (9)	0.04274 (19)	
O2	0.97405 (10)	0.21983 (10)	0.43867 (8)	0.03730 (17)	
H2A	1.078 (3)	0.201 (2)	0.4093 (18)	0.070 (5)*	
O3	0.64815 (10)	0.44948 (8)	0.32017 (7)	0.02986 (15)	
H3A	0.614 (2)	0.529 (2)	0.3718 (16)	0.055 (4)*	
O4	0.49940 (9)	0.07745 (8)	0.24744 (7)	0.02624 (14)	
H4A	0.435 (2)	0.1291 (19)	0.1931 (16)	0.046 (4)*	
O5	0.46940 (10)	0.30910 (9)	0.56376 (7)	0.03325 (16)	
O6	0.24385 (10)	0.16066 (12)	0.35901 (9)	0.0434 (2)	
O7	0.27079 (16)	0.18179 (14)	0.03248 (8)	0.0589 (3)	
N1	0.13637 (14)	0.33402 (11)	−0.18924 (9)	0.03416 (18)	
H1A	0.009 (3)	0.299 (2)	−0.2159 (17)	0.058 (4)*	
H1B	0.171 (2)	0.406 (2)	−0.2372 (17)	0.054 (4)*	
C1	0.89145 (11)	0.31305 (11)	0.36407 (9)	0.02589 (17)	
C2	0.72471 (11)	0.34681 (10)	0.40548 (9)	0.02281 (15)	
H2	0.7731	0.4103	0.5044	0.027*	
C3	0.56984 (11)	0.17709 (10)	0.38751 (8)	0.02054 (15)	
H3	0.6295	0.1105	0.4483	0.025*	
C4	0.41269 (11)	0.21858 (11)	0.44055 (9)	0.02372 (16)	
C5	0.18699 (19)	0.08025 (15)	−0.10810 (11)	0.0423 (2)	
H5A	0.0503	0.0270	−0.1283	0.051*	
H5B	0.2445	−0.0110	−0.1189	0.051*	
C6	0.21726 (18)	0.18979 (14)	−0.20995 (11)	0.0403 (2)	
H6A	0.3536	0.2358	−0.1949	0.048*	
H6B	0.1544	0.1202	−0.3058	0.048*	
C7	0.21262 (16)	0.43291 (14)	−0.04050 (12)	0.0405 (2)	
H7A	0.1471	0.5177	−0.0285	0.049*	
H7B	0.3487	0.4936	−0.0152	0.049*	
C8	0.1826 (2)	0.31178 (19)	0.05380 (12)	0.0536 (3)	
H8A	0.2377	0.3759	0.1515	0.064*	
H8B	0.0460	0.2582	0.0335	0.064*	
O1W	−0.25124 (12)	0.25085 (11)	−0.22673 (10)	0.0442 (2)	
H1W	−0.305 (3)	0.152 (3)	−0.2239 (19)	0.066 (5)*	
H2W	−0.323 (3)	0.282 (2)	−0.2912 (19)	0.061 (5)*	

### Atomic displacement parameters (Å^2^)

**Table:**

	*U*^11^	*U*^22^	*U*^33^	*U*^12^	*U*^13^	*U*^23^
O1	0.0447 (4)	0.0499 (4)	0.0573 (5)	0.0244 (4)	0.0374 (4)	0.0259 (4)
O2	0.0257 (3)	0.0513 (4)	0.0468 (4)	0.0201 (3)	0.0188 (3)	0.0194 (3)
O3	0.0370 (3)	0.0253 (3)	0.0389 (3)	0.0157 (3)	0.0226 (3)	0.0119 (3)
O4	0.0260 (3)	0.0244 (3)	0.0279 (3)	0.0086 (2)	0.0094 (2)	0.0027 (2)
O5	0.0347 (3)	0.0436 (4)	0.0309 (3)	0.0223 (3)	0.0166 (3)	0.0079 (3)
O6	0.0206 (3)	0.0625 (5)	0.0465 (4)	0.0161 (3)	0.0121 (3)	0.0031 (4)
O7	0.0882 (7)	0.0764 (6)	0.0274 (4)	0.0622 (6)	0.0101 (4)	0.0106 (4)
N1	0.0375 (4)	0.0333 (4)	0.0352 (4)	0.0091 (3)	0.0161 (3)	0.0145 (3)
C1	0.0193 (3)	0.0254 (4)	0.0323 (4)	0.0047 (3)	0.0118 (3)	0.0026 (3)
C2	0.0206 (3)	0.0224 (3)	0.0268 (4)	0.0063 (3)	0.0115 (3)	0.0035 (3)
C3	0.0182 (3)	0.0220 (3)	0.0251 (4)	0.0085 (3)	0.0104 (3)	0.0067 (3)
C4	0.0216 (3)	0.0270 (4)	0.0307 (4)	0.0122 (3)	0.0143 (3)	0.0118 (3)
C5	0.0605 (7)	0.0408 (5)	0.0332 (5)	0.0254 (5)	0.0166 (5)	0.0122 (4)
C6	0.0553 (6)	0.0387 (5)	0.0340 (5)	0.0172 (5)	0.0235 (5)	0.0083 (4)
C7	0.0359 (5)	0.0382 (5)	0.0445 (6)	0.0134 (4)	0.0125 (4)	−0.0005 (4)
C8	0.0798 (9)	0.0697 (8)	0.0315 (5)	0.0532 (7)	0.0212 (5)	0.0137 (5)
O1W	0.0343 (4)	0.0367 (4)	0.0501 (5)	0.0037 (3)	0.0014 (3)	0.0144 (4)

### Geometric parameters (Å, °)

**Table:**

O1—C1	1.2041 (11)	C2—C3	1.5310 (11)
O2—C1	1.3089 (11)	C2—H2	0.9800
O2—H2A	0.958 (19)	C3—C4	1.5367 (11)
O3—C2	1.4119 (10)	C3—H3	0.9800
O3—H3A	0.911 (17)	C5—C6	1.4976 (15)
O4—C3	1.4115 (10)	C5—H5A	0.9700
O4—H4A	0.868 (16)	C5—H5B	0.9700
O5—C4	1.2526 (11)	C6—H6A	0.9700
O6—C4	1.2425 (11)	C6—H6B	0.9700
O7—C5	1.4192 (14)	C7—C8	1.5019 (18)
O7—C8	1.4239 (14)	C7—H7A	0.9700
N1—C7	1.4803 (14)	C7—H7B	0.9700
N1—C6	1.4872 (14)	C8—H8A	0.9700
N1—H1A	0.888 (18)	C8—H8B	0.9700
N1—H1B	0.856 (17)	O1W—H1W	0.825 (19)
C1—C2	1.5224 (11)	O1W—H2W	0.848 (19)
			
C1—O2—H2A	110.4 (11)	O5—C4—C3	115.75 (7)
C2—O3—H3A	110.2 (10)	O7—C5—C6	110.47 (10)
C3—O4—H4A	109.1 (10)	O7—C5—H5A	109.6
C5—O7—C8	110.99 (9)	C6—C5—H5A	109.6
C7—N1—C6	111.80 (8)	O7—C5—H5B	109.6
C7—N1—H1A	108.3 (11)	C6—C5—H5B	109.6
C6—N1—H1A	113.0 (11)	H5A—C5—H5B	108.1
C7—N1—H1B	106.1 (10)	N1—C6—C5	109.55 (8)
C6—N1—H1B	109.1 (11)	N1—C6—H6A	109.8
H1A—N1—H1B	108.3 (15)	C5—C6—H6A	109.8
O1—C1—O2	124.30 (8)	N1—C6—H6B	109.8
O1—C1—C2	122.71 (8)	C5—C6—H6B	109.8
O2—C1—C2	112.95 (7)	H6A—C6—H6B	108.2
O3—C2—C1	108.22 (7)	N1—C7—C8	109.64 (9)
O3—C2—C3	110.63 (6)	N1—C7—H7A	109.7
C1—C2—C3	110.96 (6)	C8—C7—H7A	109.7
O3—C2—H2	109.0	N1—C7—H7B	109.7
C1—C2—H2	109.0	C8—C7—H7B	109.7
C3—C2—H2	109.0	H7A—C7—H7B	108.2
O4—C3—C2	111.41 (6)	O7—C8—C7	110.28 (10)
O4—C3—C4	113.65 (6)	O7—C8—H8A	109.6
C2—C3—C4	108.61 (6)	C7—C8—H8A	109.6
O4—C3—H3	107.6	O7—C8—H8B	109.6
C2—C3—H3	107.6	C7—C8—H8B	109.6
C4—C3—H3	107.6	H8A—C8—H8B	108.1
O6—C4—O5	126.30 (8)	H1W—O1W—H2W	110.3 (17)
O6—C4—C3	117.95 (8)		
			
O1—C1—C2—O3	1.65 (12)	C2—C3—C4—O6	125.59 (9)
O2—C1—C2—O3	179.30 (7)	O4—C3—C4—O5	−178.95 (7)
O1—C1—C2—C3	123.19 (9)	C2—C3—C4—O5	−54.34 (9)
O2—C1—C2—C3	−59.15 (10)	C8—O7—C5—C6	62.14 (15)
O3—C2—C3—O4	62.09 (8)	C7—N1—C6—C5	53.29 (13)
C1—C2—C3—O4	−58.03 (8)	O7—C5—C6—N1	−56.83 (13)
O3—C2—C3—C4	−63.83 (8)	C6—N1—C7—C8	−53.28 (12)
C1—C2—C3—C4	176.05 (7)	C5—O7—C8—C7	−61.96 (16)
O4—C3—C4—O6	0.98 (11)	N1—C7—C8—O7	56.73 (14)

### Hydrogen-bond geometry (Å, °)

**Table:**

*D*—H···*A*	*D*—H	H···*A*	*D*···*A*	*D*—H···*A*
N1—H1B···O3^i^	0.856 (17)	2.036 (17)	2.8430 (11)	156.6 (15)
N1—H1A···O1W	0.888 (18)	1.888 (18)	2.7583 (13)	166.1 (16)
O1W—H1W···O4^ii^	0.825 (19)	2.013 (19)	2.8173 (11)	164.6 (18)
O1W—H2W···O5^iii^	0.848 (19)	1.921 (19)	2.7542 (11)	167.2 (17)
O2—H2A···O6^iv^	0.958 (19)	1.584 (19)	2.5412 (10)	177.3 (17)
O3—H3A···O5^v^	0.911 (17)	1.742 (17)	2.6398 (9)	167.9 (15)
O4—H4A···O7	0.868 (16)	1.939 (16)	2.7818 (10)	163.4 (14)

Symmetry codes: (i) −*x*+1, −*y*+1, −*z*; (ii) −*x*, −*y*, −*z*; (iii) *x*−1, *y*, *z*−1; (iv) *x*+1, *y*, *z*; (v) −*x*+1, −*y*+1, −*z*+1.
